# Gastroscopy for dyspepsia: Understanding primary care and gastroenterologist mental models of practice: A cognitive task analysis approach

**DOI:** 10.1093/jcag/gwad035

**Published:** 2023-09-27

**Authors:** Tanya Barber, Katelynn Crick, Lynn Toon, Jordan Tate, Karen Kelm, Kerri Novak, Rose O Yeung, Puneeta Tandon, Daniel C Sadowski, Sander Veldhuyzen van Zanten, Denise Campbell-Scherer

**Affiliations:** Office of Lifelong Learning & the Physician Learning Program, Faculty of Medicine and Dentistry, University of Alberta, Edmonton, AB, Canada; Office of Lifelong Learning & the Physician Learning Program, Faculty of Medicine and Dentistry, University of Alberta, Edmonton, AB, Canada; Division of Nephrology, Department of Medicine, University of Alberta, Edmonton, AB, Canada; Office of Lifelong Learning & the Physician Learning Program, Faculty of Medicine and Dentistry, University of Alberta, Edmonton, AB, Canada; Department of Pediatrics, Faculty of Medicine and Dentistry, University of Alberta, Edmonton, AB, Canada; Division of Gastroenterology, Cumming School of Medicine, University of Calgary, Calgary, AB, Canada; Office of Lifelong Learning & the Physician Learning Program, Faculty of Medicine and Dentistry, University of Alberta, Edmonton, AB, Canada; Division of Endocrinology and Metabolism, Department of Medicine, University of Alberta, Edmonton, AB, Canada; Office of Lifelong Learning & the Physician Learning Program, Faculty of Medicine and Dentistry, University of Alberta, Edmonton, AB, Canada; Division of Gastroenterology, Department of Medicine, Faculty of Medicine and Dentistry, University of Alberta, Edmonton, AB, Canada; Division of Gastroenterology, Department of Medicine, Faculty of Medicine and Dentistry, University of Alberta, Edmonton, AB, Canada; Division of Gastroenterology, Department of Medicine, Faculty of Medicine and Dentistry, University of Alberta, Edmonton, AB, Canada; Office of Lifelong Learning & the Physician Learning Program, Faculty of Medicine and Dentistry, University of Alberta, Edmonton, AB, Canada; Office of Lifelong Learning & the Physician Learning Program, Faculty of Medicine and Dentistry, University of Alberta, Edmonton, AB, Canada; Department of Family Medicine, Faculty of Medicine and Dentistry, University of Alberta, Edmonton, AB, Canada

**Keywords:** gastroscopy, cognitive task analysis, mental models, referrals, practice support, dyspepsia

## Abstract

**Background:**

Gastroscopy to investigate dyspepsia without alarm symptoms rarely results in clinically actionable findings or sustained health-related quality-of-life improvements among patients aged 18–60 years and is, therefore, not recommended. Despite this, referrals for and performance of gastroscopy among this patient population remain high. The purpose of this study was to understand family physicians’ and gastroenterologists’ mental models of dyspepsia and the drivers behind referring or performing gastroscopy.

**Methods:**

Cognitive task analysis routine critical decision method interviews with family physicians (*n* = 8) and gastroenterologists (*n* = 4).

**Results:**

Family physicians and gastroenterologists hold rich mental models of dyspepsia that rely on sensemaking; however, gaps in information continuity affect their ability to plan and coordinate patient care. Drivers behind decisions to refer or perform gastroscopy were: eliminating risk for serious pathology, providing reassurance, perceived preference by patients to receive information and reassurance from gastroenterologists, maintaining relationships with patients, and saving costs to the health system.

**Conclusions:**

Family physicians refer for dyspepsia when they are seeking support from gastroenterologists, they believe that alternative factors may be impacting the patient’s health or view it as a cost-saving measure. Likewise, gastroenterologists perform gastroscopy for dyspepsia when they perceive it as a cost-saving measure, they want to support their primary care colleagues and provide their colleagues and patients with reassurance. An improved degree of communication between speciality and primary care could allow for continuity in the transfer of information about patients and reduce referrals for dyspepsia.

## Introduction

Dyspepsia is a common, usually benign condition, that is frequently investigated using gastroscopy.^1,2^ Dyspepsia involves a cluster of symptoms such as epigastric pain or discomfort, early satiety, bloating, upper abdominal fullness, or nausea.^2–6^ A substantial proportion of patients respond to treatment with a proton pump inhibitor and/or a test and treat strategy for *Helicobacter pylori*, and generally do not require a gastroscopy.^6–9^ Patients who present without alarm symptoms (e.g., vomiting, bleeding, unintended weight loss, dysphagia) are often referred for gastroscopy, despite the usually low yield of clinically actionable findings in patients <60 years of age.^5,10–17^ Even in the presence of alarm symptoms, worrisome findings on gastroscopy are rare.^18–20^

Long wait times to access speciality care and costs are a growing concern in Canada where demand continues to exceed capacity.^21,22^ Given the low yield of investigating dyspepsia symptoms among patients <60 years of age with gastroscopy, its use is discouraged by multiple guidelines including the Canadian Association of Gastroenterology and the American Gastroenterology Association.^7,8,15,17,23,24^

Despite this, gastroscopy continues to be used to investigate dyspepsia in adult patients under 60 years in 24–35 percent of cases in North America.^12,25^ Known reasons for referral by family physicians for gastroscopy include alarm symptoms, failure to respond to PPI treatment or anti-Helicobacter therapy, overlap with irritable bowel syndrome, and previous history of dyspepsia or chronic recurrent symptoms.^26^ Additionally, family physicians want to exclude serious pathology and avoid missing cancer or other worrisome, treatable causes,^27–29^ and reassure the patient.^29^ They may refer to address patient health-related anxiety^1,27,28,30^ or a perceived patient expectation that gastroscopy will be performed.^31,32^

Several useful interventions in reducing the use of gastroscopy for the investigation of dyspepsia symptoms include web-based education for patients, physician audit and feedback, nurse-led shared medical appointments, referral systems that use clinical pathways, guideline adaptation and active implementation strategies, and decision-aids.^1,33–35^

In order to select and co-design processes and interventions to help optimize the use of gastroscopy for dyspepsia, we need to understand the mental models of family physicians and gastroenterologists and identify the contextual drivers behind their decision making. Mental models determine what we pay attention to, what options and possibilities we consider, and how we make sense of events, solve problems, make decisions, and act. Ultimately, they can assist in understanding what we are willing to adopt, implement, and maintain.^36–38^ This understanding is essential to how we should adjust our approach to the co-creation of possible solutions that will apply within Alberta and potentially transfer to other health systems. While understanding the perceptions and mental models of patients living with dyspepsia would add a valuable lens for co-creating solutions, our focus was to examine the decisions made by physicians to refer or perform gastroscopy. Exploring the patient perspective was, therefore, beyond the scope of our study; however, existing literature provides an insight into these important patient perspectives.^31,39–42^ The goal of this study was to elicit family physicians’ and gastroenterologists’ mental models of referring or performing gastroscopy among patients aged 18–60 years without clear alarm symptoms and the drivers behind their decision making.

## Methods

We used an adapted cognitive task analysis (CTA) technique called the routine critical decision method, which keeps the participant grounded in a recent and routine case. CTA is a family of methods or techniques developed to elicit and represent tacit knowledge held by individuals and teams when performing “real-world” tasks in cognitively complex environments such as aviation, military command, and intensive care units.^36^ CTA has also been applied extensively in primary care.^43–47^

CTA can be used to identify and understand the mental processes, which are called macrocognitive functions, such as sensemaking, detecting problems, decision making, and managing uncertainty (Table 1), which are required in performing a task. Understanding these mental processes informs how we describe the mental models held by individuals or teams related to that task.^36,38,48–50^

**Table 1.** Macrocognitive functions framework.

Function	Description
Sensemaking and learning	Deliberate attempt to gather and share information to build a coherent, conceptual, situational understanding of an event, problem, or process Includes sense giving (presenting an understanding to others to adopt)
Decision making	Thinking around how to make decisions and what decisions should be made Decisions in, or about, patient care and administrative processes
Planning and re-planning	Making plans and adjusting as needed Shaping or reshaping patient care or administrative processes
Monitoring and problem detection	Evaluation of how things are going and what needs attention Tracking the progress or outcomes of patient care or administrative processes Planned, ad hoc (“noticing”), formal (data collection), or informal
Managing the unknown, unclear, unexpected, and irregular	How individuals or teams deal with the unknown and unexpected Planned or anticipatory (contingencies, fallbacks); Unplanned (“scrambling”) Evaluating/estimating risks
Coordinating	Any activity that helps synchronize 2 or more individuals in a patient care or administrative process, especially transmitting information or expectations Maintenance of “common ground,” shared expectations/understanding/mental models of processes

### Data collection and analysis

Our CTA-trained team members interviewed participants in pairs, one interviewer and one note taker,^36^ virtually via Zoom. Using a semi-structured interview guide (Supplementary File 1) participants were asked to consider a patient case where they, as physicians, decided to refer or perform a gastroscopy for a patient 18–60 years of age, with vague or no alarm symptoms. Participants were encouraged to use their electronic medical records or consult notes to assist with their retrospection of the case. Interviews took 60–90 minutes, were audio recorded and transcribed for analysis. No patient-identifiable information was collected, apart from age range, gender, and/or sex.

The interview transcripts were coded by all CTA-trained team members individually using a framework based on the macrocognitive functions (Table 1).^36,48–50^ Then team analysis meetings were held virtually (via Zoom) to identify key macrocognitive functions used, drivers behind their decisions, and construct mental model representations for each participant. Analysis meetings took place concurrently with data collection and allowed team members to discuss and resolve discrepancies around coding, identified macrocognitions, or descriptions of mental models. One of the two team members who conducted the interview attended the analysis session to provide opportunities for further clarification. Contrasts and comparisons across participants were compiled and categorized.

### Participants

We interviewed twelve participants in total (*n* = 8 family physicians, *n* = 4 gastroenterologists). While participants were selected purposefully^51^ to create a sample representing urban versus rural, fee-for-service or academic payment, and years of practice, the majority of our participants practised in urban settings (Table 2). Recruitment took place over three months, February–April 2022 and involved using existing relationships with colleagues to invite participation, advertising through a Facebook Group for family physicians, and posting notices via the Physician Learning Program^52^ website and the Alberta Medical Association’s (AMA) online newsletters. Participants were directed to contact a member of the CTA research team for further information, ensuring that their colleagues would not know if they agreed to participate. Participants were offered an honorarium at a lower hourly rate ($132 CDN/hour) than their usual salary or fee-for-service rates to recognize the value of their time but not coerce or influence participation. No participants withdrew from the study.

**Table 2.** Patient demographics.

	FPs (*N* = 8)	SPs (*N* = 4)
**Type of practice**		
Fee-for-Service	7	1
ARP-Academic		3
Locum (various practice types)	1	
**Years practicing**		
Under 5 years	1	
5–10 years	5	
11–20 years		3
Over 20 years	2	1
Geographic location		
Metro Northern AB	5	4
Non-Metro Northern AB	1	
Non-Metro Central AB	1	
Non-Metro Southern AB	1	

## Results

### Mental models

It is common to see differences among participants’ mental models,^47^ particularly between speciality and primary care physicians^45,46^; however, in this study, we found that there were more similarities between groups. They held rich mental models of dyspepsia, that is, their mental models included detailed conceptual elements, interconnections between concepts, and diverse options, exceptions, and limitations related to their approach to dyspepsia.^37,53^ These mental models relied heavily on sensemaking and learning (Table 1) and were primarily patient-centred.^54,55^ Participants had similar drivers behind their decisions to refer, accept, and/or perform a gastroscopy.

The differences we identified between both groups’ mental models related to the nature of their practices. For instance, gastroenterologists had more in-depth mental models of dyspepsia and gastrointestinal issues given this is their area of specialization. However, family physicians found dyspepsia was common enough that they were quite familiar with its symptoms and treatments.

Family physician mental models included a plan of care involving investigations for screening and to rule out other health issues, advising patients about lifestyle modifications, and trialling medications before deciding to refer (Table 3, section 1.1). This plan of care was based on previous knowledge learned via medical school or continuing medical education, and current guidelines (Table 3, 1.2). However, enacting this plan is effortful for family physicians who are tasked with managing both general health issues and those specifically related to dyspepsia, as well as keeping patients informed throughout the process. The effort involved is often compounded by the fact that patients may not follow the plan of care. While some clinical pathways exist to support family physicians, these can be difficult and time consuming to navigate, and may not fit within existing workflows or may compete with existing tools (Table 3, 1.3).

**Table 3.** Illustrative quotations from CTA data—family physicians (FPs) and gastroenterologists (GIs).

1.0. *Family physicians’ mental models of referring for gastroscopy in patient population under study*	
1.1. *Plan of care prior to referral*	I guess my role also would be to try to figure out if it is actually dyspepsia or some other symptom or like what exactly the symptom is. And then—try to do the relevant investigations … and then trialling medications … FP4 She was complaining of some substernal epigastric pain, I had taken a history and she did not have any alarm features, and I trialled her on PPI. … in addition to that, just doing some education around some common triggers for GERD and dyspepsia and doing kind of a medication lifestyle overview with her. … And then also did some blood work investigation … And then, a couple of weeks after being on the higher dose PPI, she was still having symptoms without much improvement. And so, at that point, I did refer her for a GI consult to ask them to consider scoping her. FP7
1.2. *Previous sense making and learning*	…it’s sort of a multi-layered learning process … in medical school, we’re… taught about what investigations are needed to make a diagnosis for certain conditions, and as you get into your clinical or your clerkship… you see other physicians …starting with a set of symptoms and seeing how they work through the process of building a differential diagnosis and choosing their investigations in order to narrow down that differential…. I would say it’s a combination of that clinical experience and training… when it comes to a common symptom, like reflux or dyspepsia. It’s something that I’ve seen so many times that it’s not as conscious of a process as building that differential diagnosis. But it’s something that I’ve seen before and —I have in my mind a list of things that need to be ruled out. FP6 I attend a lot of CMEs from specialists and, a lot of times they’ll have slides that say when to refer and they’ll tell you all your—you know, your red flags and things like that… FP1 I think the guidelines are you do them enough and then it becomes just routine. FP3
1.3. *Effort in managing plan of care*	… this lady comes in very sporadically, but with a bucket list of problems that flow one into the other…. and she’s on different medications for different things. But it makes the whole story very confusing. … And she had really been sort of pushing for scope from the very beginning, but I knew that, we’re not going to get you in without first trying proton pumps, and maybe looking at your other meds and doing some other stuff … But then she’d come back complaining of the same stuff, but she wasn’t really compliant with the meds and then in the meantime, she upped a bunch of other doses on her own for some other conditions. FP2 …there’s so many pathways. Um, have I read that specific one [provincial Alberta Health Services (AHS) Dyspepsia pathway]? Not that I recall. … I imagine it’s similar to the guidelines... Let me see if it comes up… Yeah, dyspepsia doesn’t come up at all. …[it’s] Not at my fingertips. FP6 It was actually when I saw in the email about this research study, that I was like, “Oh, I didn’t even realize that AHS had a dyspepsia pathway.” …And so I thought, “Oh, I should look at that”, and when I was looking at the pathway, I thought about this case that we’re discussing—I closely followed the pathway. But, there were a couple of the blood work tests that I didn’t think to order, like, I didn’t think about doing a coeliac screen with this patient’s particular presentation…. But in the end, with the patient not responding to a PPI and not finding another diagnosis, I still think it would be appropriate to refer. FP7
1.4. *Gaps in information from GI*	I also appreciate if a specialist gets back to me and says “While they’re waiting to be seen by me, you know, here’s a treatment to try” or “Here are some other investigations that would make the referral visit more efficient or productive”. So, having some interim advice depending on the case can be helpful. FP7 I guess also offering suggestions…Giving me some medication advice, perhaps to sort of help with her symptoms if the scope was negative…. FP4 …it’s clear from the referral letter, what you’re referring for, they’ll send back a whole host of suggestions, say, “Try this, try this, try this”. Fabulous, happy to do that, you know, because then I can say, “Listen, the specialist, you know, read this letter, and has given us some suggestions to try, either while you’re waiting for your procedure, or instead of the procedure”. It just kind of takes some of the heat off of me. FP6
1.6. *Decision making and urgency of referral*	…I’ll say “I’ll send you to the surgeon about X or Y and they may or may not want to operate on you or they may or may not want to provide you this therapy, but I think they should be the one to decide what that would be”. FP5 “I appreciate your consideration of an upper scope”. Like if the specialist can sit down and talk to the patient and talk them out of it. Great. I have no issue with that. FP6 And so, if I think something needs to be dealt with more urgently, then I would try to call a physician and let them know, I’m sending a referral to you…. I would try to phone the specialist to let them know so that they can make a decision whether or not the patient needs to be seen sooner. FP7
2.0. *Gastroenterologists’ mental models & approaches of accepting referrals for and performing gastroscopy in patient population under study*	
2.1. *Sense making and learning from a “good” referral letter*	I look at every single referral that comes through… if—it’s vague history, or there’s incomplete information, I usually bounce those back immediately. I think in a case like this, because it was so well packaged together and followed really good referral criteria and laid a good story, but not a five-page letter… point form, things were done correctly—you could tell you know, it was short and succinct, but it was very clear what was happening. SP2 …on average, the referral letters we get are two sentences. So, something that’s in paragraph form is helpful…it says, “Here’s what I have tried empirically for this patient”, and sometimes includes a little bit of their past history or for this patient who had a complex social history, it included some of those features. So, …that you can see that they’ve gone through the pathways already and given every attempt and now they’re just kind of looking for some additional support. It’s uncanny how often we get someone who has not even tried PPI as a referral to a specialist. SP4
2.2. *Understanding family physicians’ context and their ability to manage dyspepsia with support*	The thing that that I think specialists forget, is that a lot of patients with functional dyspepsia, they come to see their family doctors often. And, you know, that can be exhausting to family physicians, and they’ve tried everything, and they don’t know what else to do. And so, when they refer, you know, it’s a cry for help for themselves, not just for the patient. And usually, those are the people who’d write really nice letters. Like, this is what we’ve been through, this is what I tried what I was worried about, but I’m at my wits’ end. Okay. And I honestly think that those are the patients we should see. And again, I’m not talking red flags, like I’m talking like your average dyspepsia person. SP1 I mean, the family doctors are good. They just need a bit of guidance from us and saves everybody time. … the family physician had done all the appropriate bloodwork and tests. So, this was really the last test I think that was necessary to give reassurance. …oftentimes, reassurance from the family doctor is not enough for the patient…I feel that patients often need that interaction with a specialist. SP3
*3.0. Key drivers for referring/performing gastroscopy in patient population under study—Reassurance & Ruling out Risk*	
3.1*. Reassurance & ruling out risk for patients & physicians*	I wasn’t particularly worried that there was some, you know, really sinister thing going on, because she had had this issue for so long and no red flags. But I was hoping for her that she would get some either reassurance or, you know, some other method of treatment for her symptoms that I wasn’t as familiar with, from the GI … I was hoping she would get some relief and then also reassurance. FP1 “Are you worried about cancer?”[patient asked], I said, “No, I just want to know that I’m not missing anything”. FP8 I think there’s enough there to say, “You know what, I probably need to make sure that she’s not one of that 5%, that actually has something significant”. … we’re always worried about looking for cancers, but there’s other things that cause discomfort, you know, people with anatomic defects, large hernias … SP2 …my goal is to make sure we’re not missing something else, —get a differential and then to actually examine the patient, and then just have a discussion with them about what dyspepsia is, and also talk to them about endoscopy and letting them know there are risks and benefits … So, I also think that when patients actually sit down with a specialist and have that conversation, that itself gives us—gives them reassurance. SP3
3.2. *Patient’s push for referral and “needing to hear it from a specialist”*	I think the family doctors are also getting frustrated because they’ve done everything they can. And oftentimes, reassurance from the family doctor is not enough for the patient… I feel that patients often need that interaction with a specialist. SP3 The other thing that I think will help patients is to say, I talked to a gastroenterologist, I went over the story, your story with the gastro, and he or she did not think it needed to be seen. I think for the family docs that’s also a very powerful piece, because they’re getting pressured by patients, obviously, right. I need to be seen, I need to be seen. So, you know, “Mrs. X, I talked [about] your case with a GI and the GI listened, —and they felt at the end, no, you didn’t need to be seen”. SP2 I would actually show her [patient] the UpToDate screen and say, “well, there’s nothing overly alarming here, like I really don’t think they’ll see anything on scope”. FP2 So, when patients come in and ask for something when they do not have enough grounds, … if they still are, you know insisting on it, I can do the referral, and put in ‘patient is insisting on getting a colonoscopy or an endoscopy for this reason, my clinical grounds’—I state them. … Because the patient wanted it, there was no clinical ground. My consultant colleague said, no, absolutely agree…Patient doesn’t need to be seen for these reasons. When I go back to them and say, “You know what, I did it as you told me to and—it was rejected for these reasons. Patients are - I think they’re happier that way, I said something validated by the specialists, they’re not worried anymore. 90 percent of time it works well. FP8 And so sometimes—I do try to be clear in my referral letters just if I’m asking for advice about how a patient should be investigated. And sometimes I’ll say, “I don’t know that this patient needs to be seen by you in person, but they would be happy to see you if you feel that would be appropriate. Otherwise, please provide any recommendations for how we can investigate or manage this”. FP7
4.0. *Key drivers for referring/performing gastroscopy in patient population under study—Building & maintaining the Therapeutic Relationship*	
4.1. *Refer or accept referral to build & maintaining the Therapeutic Relationship*	So, they [patient] start to get worried, or their wife [laughter] in the case of this gentleman, his wife is very anxious. She’s also my patient. … I think then, it is hard to talk people off the ledge and you risk losing that therapeutic relationship with that patient or that family. If you refuse something that actually seems fairly reasonable, and they are struggling with symptoms, and you don’t have [a complete] answer…. FP6 …a pretty complicated patient with a lot of like psych overlay… the things that have been going through my head are both a mix of trying to keep rapport with the patient, while trying to get them to the care they need, but not push them too far. FP5 It’s a process for them [patients] to get to trust you as well…So, I think those are the people that, you’re not worried that there’s anything bad going on, they’ve had a lot of tests, and you’re trying to build the relationship, and kind of begin the process of explaining what’s going on so that hopefully at future follow-ups…they get a better sense of … not just reassurance but an empowerment… SP1 So, I have to say the consultation I don’t think changed my expectation of what we would be doing next, which was some symptomatic therapy and referral to endoscopy. But I think from a patient communication point of view, it was important to build rapport and to get a fuller picture of the patient. SP4
4.2. *Use of trust and rapport to encourage patient to accept referral*	So, he was quite resistant to seeing GI because he felt that they will take away his meds or give him a very, very serious diagnosis that he wasn’t ready to take. …[participant told patient] “You know what, I will send you to someone I would go to. So, I trust them. I know them well enough. I know their practice. Nobody’s there to hurt you. We just want to do the right thing.” FP8
5.0. *Key drivers for referring/performing gastroscopy in patient population under study- Saving Costs to healthcare system*	
5.1.*Saving Costs to healthcare system*	…honestly, these patients, I’ve been doing this long enough to know that if they can just talk to a specialist themselves, even if the specialist isn’t telling them anything differently than what I’ve told them, there is a power—and I’m not going to say placebo, because maybe it’s not just placebo. But who cares if it’s just placebo? If it works, it’s actually cheaper for the healthcare system to just have the patient talk to a specialist, and then it’s over. —as opposed to continually coming back to me—I mean I charge a lot of money—repeated visits like that to healthcare system, right? FP2 I tried to emphasize the fact that she has no red flags, which is reassuring, but I know some patients are very anxious. And so, I’ve also had some other patients that have had sort of abdominal pain or other symptoms that just kind of present themselves in the emergency department … FP4 … a family doctor who is aware of the pathways and is trying to manage things in a non-specialist way. And instead they [patient] will go to a walk-in clinic where they think that they can get a referral, and they do…. which would be so frustrating as the family doctor who’s doing everything right. SP4 Oftentimes, patients go to the emergency room because either [they’re] frustrated and I mean they have seen their GP…—and probably 9.5 times out of 10, the scope is going to be normal. But now, once they’ve been scoped, then we can say, this patient has been thoroughly investigated. Everyone’s reassured. … It’s [recurrent emergency visits] a big, expensive stress on the system…. So, in my mind, I’m thinking, “Okay, how many more times are they going to be in the emergency room?”. SP3

Gaps in coordination, sensemaking, and informational continuity (i.e., the communication of key facts and opinions across teams, institutions, providers, and between providers and patients)^56^ were noted by participants and are key to understanding what is desired for improved care. Family physicians indicated that they lacked information from gastroenterologists such as recommendations or plans of care for patients with dyspepsia and would appreciate receiving this information at any point of the patient’s journey (Table 3, 1.4). They indicated that their referral was not necessarily to book a direct gastroscopy procedure, but rather to request a gastroenterologist consultation with the patient to provide information, alternatives, offer reassurance, and further education. If family physicians felt strongly that the patient urgently needed a gastroscopy, they would call the specialist directly (Table 3, 1.5).

The gastroenterologists we interviewed also indicated gaps in coordination and informational continuity. They reported that they often receive referral letters with incomplete information as to why gastroscopy is indicated in patients aged <60 with no alarm symptoms. They require more information from family physicians to make fully informed decisions. Their sensemaking, learning, and decision making (Table 1) for accepting a referral were highly dependent on what they called a “good” referral letter (Table 3, 2.1). In cases where family physicians had provided details, the gastroenterologists were aware of the constraints facing their primary care colleagues. Part of their mental models was the belief that family physicians can manage dyspepsia with support from speciality care. They recognized that family physicians may refer patients with no or unclear alarm symptoms because the family physician was seeking support, having exhausted other options and possibilities, and/or because patients needed specialist reassurance (Table 3, 2.2).

### Drivers decision making

A key aspect across all mental models and a key driver behind decision making was reassurance and ruling out risk. Both gastroenterologists and family physicians indicated their decisions to refer or perform a gastroscopy were shaped by the need to reassure the patient and themselves that they had ruled out any risk of finding something serious or actionable, for example, cancer. Both felt the results of a gastroscopy would reassure the patient that their health issue was functional dyspepsia, that is, no organic cause was found for the symptoms, which could be managed with lifestyle modifications and varying use of medications and reassurance. They discussed at length the anxiety and psycho-social elements that may be taking place with patients and how referring or performing a gastroscopy may alleviate concerns and anxiety (Table 3, 3.1).

Both gastroenterologists and family physicians felt patients needed to hear from a gastroenterologist to feel reassured. Family physicians explained that patients may not accept information or plans of care until they have been evaluated by a gastroenterologist. The family physicians described using the following strategies to address this: (1) showing patients clinical pathways, so the patient could see what steps are needed to be completed prior to referral; (2) using provincial services to link to an on-call specialist to ask questions and relay information to the patient; (3) referring to the gastroenterologist indicating the need for gastroscopy was unlikely, however, the patient was requesting it, with the hope that the referral would be rejected yet the patient would be provided with the reassurance they tried; and (4) referring the patient but hoping the gastroenterologist would see the patient for a consultation rather than directly to gastroscopy (Table 3, 3.2).

Gastroenterologists and family physicians commented on the importance of their therapeutic relationship with patients, and how the need to build and maintain trust also influenced their decisions around referring or performing gastroscopies for dyspepsia. Family physicians felt that if they did not pursue a referral, it could jeopardize their relationship with the patient, who may then decide to stop seeking care (Table 3, 4.1). One family physician discussed a case where she felt the patient did need a referral but had to handle it sensitively, relying on the trust she had built, as the patient was reluctant to see any other health providers (Table 3, 4.2).

For some participants, the decision to refer or perform a gastroscopy was linked to saving the health system the cost of recurring visits with the patient. Family physicians described patients who felt anxious about their symptoms and thus repeatedly booked visits, pushed for referral, or would go to other clinics or the emergency department in order to get the tests they wanted, thus working around the typical steps of investigations and care planning. Physicians noted the cost of one consultation or gastroscopy was likely less than the time and resources these patients used in primary care, even if reassurance was short term. In addition, gastroenterologists explained that the cost of a patient’s numerous repeat visits to emergency departments was a significant factor in their decision to perform a gastroscopy (Table 3, 5.1).

## Discussion

The family physicians and gastroenterologists we interviewed have rich mental models of dyspepsia and consider similar drivers in their decision making to refer, or perform, gastroscopy. The key driver is to provide reassurance to patients and physicians that the risk for a serious pathology has been eliminated and validation that the symptoms fit a functional dyspepsia diagnosis. These findings align with previous studies that reviewed models of reassurance, often with contradictory findings on whether gastroscopy and other such procedures provide a long-term solution.^26–29,41,57–59^ Some of the uncertainty, as to the degree a procedure or consultation can provide reassurance, may be related to whether implicit (specific to health concerns of patient) or explicit (generic statements) reassurances are provided to the patient, or the patient’s level of health anxiety,^58^ emotional distress about the illness,^59^ and psychological risk.^41^ Another factor is whether positive, understandable explanations for symptoms and test results, which acknowledge the patient’s concerns, were provided to patients, which specialist consultations can offer.^58,60^ Despite participants being aware of the low risk for serious pathology, and that the gastroscopy may not provide long-term reassurance, they still felt it was worth referring or performing gastroscopy since not proceeding with these actions may jeopardize their patient relationship.

There were participants who felt that referring or performing gastroscopy could save the health system costs of recurring primary care or emergency visits. While the evidence shows that gastroscopy among this patient population is not cost effective in terms of the detection of malignancies or other treatable, underlying organic causes,^12,15–18,61–64^ there is a debate about whether there is a therapeutic benefit of a negative gastroscopy result and resulting cost-savings from reduced healthcare utilization among some patients. Some studies report that following a gastroscopy there are increases in patient satisfaction, reductions in healthcare utilization, and improvements in patients’ health-related anxiety.^30,65,66^ Other studies have shown no long-term, sustained changes in healthcare utilization, reassurance, symptom improvement, or health-related quality-of-life improvement.^29,42,58^ There is some evidence that addressing and treating health-related anxiety may be less costly, and equally or more effective than performing gastroscopy to achieve these same aims.^67^ These discrepancies, and the cognitive complexity of weighing these different factors when making gastroscopy decisions for these patients, may contribute to the overuse of gastroscopy despite recommendations against its use. More comparative research may need to be completed on the costs of multiple visits to primary care and emergency, and performing the gastroscopy. In addition, studies exploring the mental models of fee-for-service gastroenterologists may provide other insights into perceptions of cost-benefits for gastroscopy for dyspepsia.

Our results indicate a need to increase coordination and information sharing between gastroenterologists and family physicians in the forms of guidance on other tests to order, medications to trial, behavioural suggestions, or a general plan of care pre- and/or post-gastroscopy. Implementable methods to share this information could include using consult notes, rejection letters, result notices, or virtual or tele-services that connect the physicians directly. Improved communication may provide a mechanism for offering a level of reassurance to patients and physicians. The Digestive Health Strategic Clinical Network, created to advance health improvements and foster sustainable solutions in digestive health,^68^ developed a primary care dyspepsia pathway to hep standardize management and potentially decrease demand for low-yield gastroscopy.^69^ Care pathways, such as the dyspepsia clinical care pathway, can vary in uptake and use, often due to issues with accessibility and lack of input from primary care, including the context within which physicians work.^70–72^ Understanding how family physicians make decisions and approach their work provides opportunities to incorporate this knowledge into existing pathways, such as adjusting for access, how it fits with clinic work-flow and existing mental models, and what and how information is provided. In addition, new pathways can be codesigned with this knowledge from the outset.^70–72^ Recently, the Physician Learning Program^52^ developed a series of “Audit and Feedback” sessions for gastroenterologists on patients who are referred for dyspepsia but without alarm symptoms, based on these CTA findings and highlighting the drivers of reassurance and cost-savings supported by the literature.^29,33,64,73^ Gastroenterologists attending these sessions agreed that committing to providing more guidance was a feasible and implementable action to increase information continuity and coordination with primary care. These actionable strategies were developed within the Alberta context; however, we hope readers will see ways in which these actions could transfer to their own practices across Canada or beyond, whether it be including more information in consult notes or reviewing and adapting clinical care pathways to work within primary care.

## Limitations

We recognized during our analysis that we were missing representation from two groups that could offer different mental models: fee-for-service gastroenterologists, and family physicians who work in walk-in clinics or were deemed ‘quick to refer’ by colleagues. We did try further recruitment for these groups but were unable to secure any further participants. We are also missing strong representation from rural and southern parts of Alberta, which may limit transferability to physicians in different geographical contexts. While our study has a small sample size, this is common for CTA studies with physicians,^44–46^ and our aim was not to reach saturation but rather to achieve sufficient and meaningful data to develop and compare mental models, as well as identify drivers behind low-yield gastroscopies for dyspepsia in the absence of red flags. This study successfully provides actionable knowledge through a deeper understanding of the ways in which family physicians and gastroenterologists approach decision making when referring or performing gastroscopies for patients 18–60 years of age without alarm symptoms.

## Conclusions

Understanding the mental models of dyspepsia held by family physicians and gastroenterologists has provided insight into how both groups are looking to reassure patients, but also themselves, that there is no risk for serious pathology beyond functional dyspepsia, which can be managed within primary care. Gastroenterologists offering more guidance for patients and family physicians working toward a diagnosis of functional dyspepsia is one way to increase informational continuity and address the need for reassurance while maintaining therapeutic relationships. Considering fewer referrals direct to gastroscopy, and instead, gastroenterologist consultations may also assist in decreasing low-yield endoscopy. Our findings, like other studies, confirm that reassurance remains the primary driver of referring for or performing gastroscopy for the investigation of dyspepsia in patients 18–60, indicating that effective strategies to provide this reassurance in other ways are needed. Further research examining the perspectives and mental models of both patients and physicians may add additional insights into codesigning such strategies.

## Supplementary Material

gwad035_suppl_Supplementary_File_1

## Data Availability

The qualitative data used to support the findings of this study, beyond use of selected quotations, are restricted by the Research Ethics Board at the University of Alberta to protect participant privacy.
