# Supplementary material for: Gastroscopy for dyspepsia: Understanding primary care and gastroenterologist mental models of practice: A cognitive task analysis approach
Source: J Can Assoc Gastroenterol. 2023 Sep 27;6(6):234–43. doi: 10.1093/jcag/gwad035 (PMC10723936; doi:10.1093/jcag/gwad035)
Supplement: gwad035_suppl_Supplementary_File_1 [file gwad035_suppl_supplementary_file_1.docx]

Supplementary File 2: Interview Guide/Probes

# **Specialist Interview Guide**

# These probes should serve only as a general interview guide. They are intended for use as a memory aid by team members trained in Cognitive Task Analysis. They are not to be asked verbatim or in order, nor are all necessarily expected. Depending on the interviewee and the information gained in other interviews, other probes may arise.

# Interview Guide: RcDM referral to upper endoscopy

| **Introduction** |
| --- |
| - **Thank you** for joining me for this conversation - **We’re here today to** understand your thinking and decision-making around referrals for endoscopy for dyspepsia - Our **purpose** is to gain a good understanding of how you think about and make decisions about referrals in a specific context, this is not to evaluate or test your approach, we want to gain a better understanding of what you consider, what your contextual constraints may be, what your role is in the process   - We’re going to ask you to think about a specific case where you were referred a person with dyspepsia where alarm symptoms were either not clear or absent.   - Please focus on **what happened in the actual case**, not what you would do in general.   - Our conversation is confidential, we will not share with your team what you said, nor will your clinic be identified. |
| **How this works…** |
| We’ll have you start from beginning: **when you first received the referral to the time when you booked the patient for their scope** – explaining your thinking as you go and sticking to what happened in the actual case, not what you would do in general or what you'd advise a resident to do – please feel free to reference the EMR if needed |

| **Recent Case - Timeline** | |
| --- | --- |
| **Let’s get started:**   - We’re going to think about a case that came to you directly (not through Sharp-GI, CAT), where you saw reasons to triage for an endoscopy for dyspepsia. This would be a person 18-60 years of age, where the alarm symptoms were not clear or absent, where there was a grey area. - What was the patient's gender and approximate age – don’t tell us their name - To help us stay on track – I’m going to draw this out on a piece of paper. - **Thinking of this particular patient** – **walk us through in broad terms** the different steps between the referral was received all the way to booking the scope? Who did you need to talk to, what team members were involved, processes, communications. - When was the referral received? How much time went by between the referral and the accept-reject and seeing the patient? - What was the date of the appointment scheduled and when was the scope scheduled? - Who was involved? | |
| **Deepening – what happened in this visit?** | |
| **Scanning/screening the referral:**   - When you saw this **referral**, what went through your mind? - Reading the referral note from the GP – what did you think? - Considering the symptoms – what went through your mind? - What knowledge, experiences, information did you draw on? - Were you reminded of another case? - What did you imagine might unfold with this particular case with these particular symptoms? - What options did you consider in this case? - Were there any **other options** available to you to handle this referral? (consult first, straight to scope, feedback to GP?) - What kinds of consequences did you expect from the different options? - - What let you know that the decision to accept the referral (see the patient for a consult and/or scope) would be the right to do in this case? How was this **option chosen and others rejected**?   (guidelines, evidence, tool, previous training, previous case etc.)   - Did your **thinking change** at any point, why? (flip?) - Was there any pressure in making this decision? (patient, time, capacity/waitlist, possible consequences, financial?)   **If GI saw patient for a consultation:**   - **When you entered the room**, what did you notice? What happened? - **What did you do first, second, last?** - What **concerns**, goals, or other considerations did you ask the patient about?   - Did you feel like you had to **negotiate** with the patient?  - **How did you know what the patient** wanted or needed?   - Was there any **additional information** you went to get?   - sources other than the patient? Labs, guidelines  - contacted GP?  - in this case – did you talk to **any colleagues** or team members?  - was there any time pressure?   - What was the most important thing you wanted to accomplish with the consultation? - How did you decide to scope? - What **helped you make that judgement**? - Were you **worried** or concerned about anything? If so, why? - At this point, were there any **other options** available to you or the patient?   - How was the scope **option chosen and others rejected**?  - Did your **thinking change** at any point, why? (flip?)  - How did you know that was the **right thing to do**? (guidelines, evidence, tool, previous training, previous case etc.)   - How did the **patient respond** to the decision? - Was happy with it? Concerns? - What else happened in this visit? - **What happened next**? - How did you **communicate back to the GP**? - What **information was passed in each direction**? How many iterations were needed? - In this case was there any **challenges**? What did you do to overcome them? - **How did you know the referral/consultation had gone well**? | Expectations, cues, communication? |
|  |  |
|  | Sense-making, rules? |
|  | Negotiation, options? sense-making |
|  | Sensemaking, learning? Mental model |
|  | Decision making |
|  | Mental model? |
|  | Options? Risks? cautions? |
|  | Unknown, risks? |
|  | Referral decision |
|  | expectations, mental model, goals |
|  | Coordination |
|  | Coordination, planning/re-planning, managing unknown |
| **What if…** |  |
| What if (X) has been different?   - If patient had been different in any way?   - What if patient was anxious or pushed for a scope? - If particular symptoms had been different? If alarm symptoms are absent? - If information coming from the GP had been different? If a GP calls your directly? - If your practice was a fee-for-service/academic? - When would you have decided differently? - What if you had rejected this referral? What do you imagine consequences could be? For yourself, for the patient, for the GP? - What if something would have been different in your practice? For example, your waitlist had been much longer? How did Covid change things? - Is the case we talk about typical? Remind you of previous cases? Was this case in any way similar or very different to previous cases? - If the referral had come through central triage? - (If it had been a very junior colleague scanning this referral?) |  |

| **Going more global now...** | |
| --- | --- |
| *Try to gauge how this person’s experiences have impacted their understanding and how they chose to act in the real world (why do you do what you do? how has it changed overtime?)*   - How often does this situation (referring when alarm symptoms not clear) happen for you?   - What do you usually do in these situations (when alarm symptoms are not clear/absent). - I’m aware there is central triage options – what are challenges and benefits of this option? - How do you see the **relationship b**etween primary care providers and GI specialists when it comes to referrals? - What do specialists consider when scanning referrals and making decisions? - What do you perceive to be **your responsibility** in the referral process? - What do you **believe the family physician should be doing**? What do you think their role is and should be? How do you believe they approach their work in the referral process? **Why do you think they do things the way they do?**  (How do you think other specialist approach this and why?) - What do you think patients want or expect? - What are some challenges you face? - What are some of the **work-arounds you have developed** to overcome challenges? | |
| **Closing** |  |
| - We appreciate your time and participation - Do you have any questions? |  |

**Family Physician Interview Guide**

# These probes should serve only as a general interview guide. They are intended for use as a memory aid by team members trained in Cognitive Task Analysis. They are not to be asked verbatim or in order, nor are all necessarily expected. Depending on the interviewee and the information gained in other interviews, other probes may arise.

# Interview Guide: RcDM referral to upper endoscopy

| **Introduction** | | |
| --- | --- | --- |
| - **Thank you** for joining us for this conversation - **We’re here today to** understand your thinking and decision-making around referrals to endoscopy for dyspepsia, especially when alarm symptoms may not be clear or straight forward. - Our **purpose** is to gain a good understanding of how you do referrals for dyspepsia, this is not to evaluate or test your approach, we want to gain a better understanding of how you think about it, what you consider, what your role is in the process   - We’ll be asking you about a specific case where you referred a patient with dyspepsia for endoscopy when alarm symptoms were not clear– as common of a case as this can be for your practice (typical for patients)   - Please focus on **what happened in the actual case**, not what you would do in general.   - Our conversation is confidential, we will not share with your team what you said, nor will your clinic be identified. | | |
| **How this works…** | | |
| We’ll have you start from beginning: **when you first saw the patient for dyspepsia to the time when the patient had their consultation with specialist** – explaining your thinking as you go and sticking to what happened in the actual case, not what you would do in general or what you'd advise a resident to do – please feel free to reference the EMR if needed | | |
| **Recent Case - Timeline** | | |
| **Let’s get started:**   - Think about a recent case where you referred a patient with dyspepsia, who was between 18 and 60 years of age to endoscopy, where alarm symptoms were not clear. - What was the patient's gender and approximate age – don’t tell us their name - To help us stay on track – I’m going to draw this out on a piece of paper. - **Thinking of this particular patient** – **walk us through in broad terms** what led up to the decision to refer to endoscopy and what needed to be done all the way to the patient’s consultation with the Gastroenterologist? Include patient interactions, interactions with team members, interactions with specialty, processes, communications. - When was the referral sent? - How much time went by between when the referral was sent and you received a notification of acceptance or rejection of the referral? - If accepted what date was the appointment scheduled and what date was it scheduled for? - Who was involved? | | |
| **Deepening – what happened in this visit?** | | |
| - When you saw this **patient on your schedule for the day**, what went through your mind? - **When you entered the room**, what did you notice? What happened? - What **concerns**, goals, or other considerations did you ask the patient about? - What did the patient focus on, what do you think was on their mind?   - Did you feel like you had to **negotiate** with the patient?  - **How did you know what the patient** wanted or needed?   - Was there any **additional information** you went to get?   - Were you drawing on previous knowledge of the patient?  - sources other than the patient? Labs, guidelines   - Considering the symptoms – what went through your mind? - What knowledge, experiences, information did you draw on? - Were you reminded of another case? - What did you imagine might unfold with this particular case with these particular symptoms? - Were there any open questions? Do you talk to any colleague about this case? - What options did you consider in this case? - Were there any **other options** available to you or the patient? (PPIs, H-pylori testing?) - What kinds of consequences did you expect from the different options?   - What let you know that the decision for a referral would be the right to do in this case? (guidelines, evidence, tool, previous training, previous case etc.)  - Did your **thinking change** at any point, why? (flip?)   - How was this **option chosen and others rejected**? - Was there any pressure in making this decision? (patient, time, possible consequences, others?)      - How did you decide where to refer this patient to? (central access or specific GI?)   What **helped you make that judgement**?  - what information, and knowledge, experience did you draw on?  - in this case – did you talk to **any colleagues** or team members?  - was there any time pressure? Or consideration of waitlists?   - Were you **worried** or concerned about anything? If so, why? - How did the **patient respond** to the referral? - Was happy with it? Concerns? - What else happened in this visit? - **What happened next**? - How did you **communicate the referral to the specialist/central access**? - What was the most important thing you wanted to accomplish with the referral letter? (this aims to find out the expectation: consult? Second opinion? Scope?) - Did you know **what the specialists needed** in the referral? How? - How did you know the specialist **received** the information? - What **information was passed in each direction**? How many iterations were needed? - How did you communicate the referral to your **team members**? - How was the **status of referral followed**? (where it was in process, notifying the patient, right until the appointment is complete and how the information is communicated back to primary care / received from specialty care?) - In this case was there any **challenges**? What did you do to overcome them? - If referral was sent **back/rejected**: what were the reasons given? What did you do? - **How did you know the referral had gone well**? | | Expectations, cues, communication? |
|  |  |  |
|  |  | Sense-making, rules? |
|  |  | Negotiation, options? sense-making |
|  |  | Sensemaking, learning? Mental model |
|  |  | Decision making |
|  |  | Mental model? |
|  |  | Options? Risks? cautions? |
|  |  | Unknown, risks? |
|  |  | Referral decision |
|  |  | expectations, mental model, goals |
|  |  | Coordination |
|  |  | Coordination, planning/re-planning, managing unknown |
| **What if…** | |  |
| What if (X) had been different?   - If symptoms had been different/alarm symptoms absent? - If this referral had been rejected? - What if you had chosen to refer through central access/ through a specific GI? - What if you had the option to call a specialist to discuss? - If patient had been different in any way?   - What if patient was anxious or pushed for a scope? - If it had been a very junior colleague who needed to refer this patient? - When would you have decided differently? - What if the wait time had been much longer? - Is the case we talk about typical? Typical? Remind you of previous cases? Was this case in any way similar or very different to previous cases? | |  |
| **Going more global now...** | | |
| *Try to gauge how this person’s experiences have impacted their understanding and how they chose to act in the real world (why do you do what you do? how has it changed overtime?)*   - How often does this situation (referring when alarm symptoms not clear) happen for you?   - What do you usually do in these situations? - How do you see the **relationship b**etween primary care providers and specialists when it comes to referrals to endoscopy that may not be straightforward? - What do family physicians consider? - What do you perceive to be **your responsibility** in the referral process? - What do you **believe the specialist should be doing**? How do you believe they approach their work in the referral process? **Why do you think they do things the way they do?** - How do you **choose different options for referring: referring to central access or to a hospital or to a specific GI**? (Question may lead to any existing working relationships and if they are not choosing certain specialists) 🡪 Do you avoid a certain way of referring, or a certain specialist? Why? - **For central access**: how do you perceive the system, what benefits or challenges do you encounter? Is there any feedback options when referrals are rejected? - Have you heard of Connect MD/SpecialistLink? What do you think about this option? - What do you think patients want or expect? - What are some challenges you face? - What are some of the **work-arounds you have developed** to overcome challenges? | | |
| **Closing** |  | |
| - We appreciate your time and participation - Do you have any questions? |  | |
